# Designing new strategy for controlling DNA orientation in biosensors

**DOI:** 10.1038/srep14415

**Published:** 2015-09-24

**Authors:** Chao Feng, Hong-ming Ding, Chun-lai Ren, Yu-qiang Ma

**Affiliations:** 1 National Laboratory of Solid State Microstructures and Department of Physics, Nanjing University, Nanjing 210093, China; 2 Collaborative Innovation Center of Advanced Microstructures, Nanjing University, Nanjing, 210093, China; 3 Center for Soft Condensed Matter Physics and Interdisciplinary Research, Soochow University, Suzhou 215006, China

## Abstract

Orientation controllable DNA biosensors hold great application potentials in recognizing small molecules and detecting DNA hybridization. Though electric field is usually used to control the orientation of DNA molecules, it is also of great importance and significance to seek for other triggered methods to control the DNA orientation. Here, we design a new strategy for controlling DNA orientation in biosensors. The main idea is to copolymerize DNA molecules with responsive polymers that can show swelling/deswelling transitions due to the change of external stimuli, and then graft the copolymers onto an uncharged substrate. In order to highlight the responsive characteristic, we take thermo-responsive polymers as an example, and reveal multi-responsive behavior and the underlying molecular mechanism of the DNA orientation by combining dissipative particle dynamics simulation and molecular theory. Since swelling/deswelling transitions can be also realized by using other stimuli-responsive (like pH and light) polymers, the present strategy is universal, which can enrich the methods of controlling DNA orientation and may assist with the design of the next generation of biosensors.

Biosensors, which transduce a bio-recognition event into measurable electronic or opto-electronic signal, play a crucial role in a wide range of applications, including clinical diagnosis, environmental monitoring, forensic analysis and antiterrorism^1^,^2^,^3^,^4^,^5^. As one of the most important biosensors, double-stranded (ds) DNA sensors have been studied extensively by electrochemical methods, where controlling orientation is of great significance^6^,^7^. Previous results have shown that the orientation of end-tethered dsDNA on metal substrate can be controlled by alternating current (AC) electric field, while single-stranded DNA (ssDNA) cannot^8^,^9^. Based on the different behaviors of ssDNA and dsDNA, DNA hybridization can be identified^10^,^11^,^12^. Moreover, dsDNA orientation switching dynamics in electric field will become slow when bound with biomarkers, such as nucleic acids, small molecules, ions, and proteins. The detection and size analysis of these biomarkers can thus be achieved on the basis of this property^13^,^14^,^15^,^16^.

The application of electric fields provides an especially powerful route in the operation of static surface systems as electronic signals can be rapid and localized to within a few nanometers from the electrode surface^8^. Therefore, the use of electric field for controlling the DNA orientation has been extensively studied and applied in a number of novel systems^17^,^18^,^19^. Despite this, one interesting problem occurs: how can we control DNA orientation by other methods? Actually, apart from the electric stimulus, there are many other stimuli in biological systems. For example, temperature stimulus, which is one of the most studied stimuli for the controlled drug delivery^20^,^21^. Especially, poly (N-isopropylacrylamide) (PNIPAm), with the lower critical solution temperature (LCST) of 32 °C, is the most suitable temperature-sensitive polymer in these systems^22^. However, to our best knowledge, there is nearly no study on using temperature stimuli to control DNA orientation in biosensors.

In this work, we propose a new strategy to control the orientation of DNA molecules in biosensors, namely, by copolymerizing DNA molecules with responsive polymer and then grafting the copolymer onto an uncharged substrate. In particular, the polymer can undergo swelling/deswelling transitions via altering external stimulus strength. To illustrate the responsive property, here we use a general thermo-responsive polymer and PNIPAm in dissipative particle dynamics (DPD) simulation and molecular theory, respectively. As we will show below, the DNA orientation can show dual- and triple-thermo-responsive behaviors under different polymer lengths. Further, we will also reveal the underlying physical mechanism of the responsive behaviors in our new system.

## Results

### Idea of temperature-sensitive DNA orientation design

**Figure 1:**
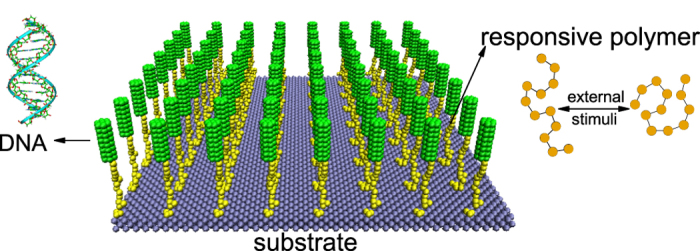
Schematic illustration of the model in simulations. The DNA molecules are copolymerized with responsive polymers that can show swelling/deswelling transitions under external stimulus and then grafted onto an uncharged substrate. Green beads represent DNA molecules, yellow beads stand for the thermo-responsive polymers, while the planar substrate is formed of the cyan beads.

Fig. 1 shows the coarse-grained models of different components in our simulations. The substrate is fabricated by arranging hydrophilic DPD beads (P) on a face-centered-cubic (fcc) lattice with lattice constant *α* = 0.50 nm^23^. Since the potential in DPD is soft-repulsive, the substrate is composed of three layers to avoid the permeation of other beads across it^24^,^25^. The DNA molecules used here are double-stranded ones with the length of 4.0 nm (~12 bp), according to the practical biosensor, where 12 bp dsDNA are used to detect protein and small molecule analytes^26^. In theories and simulations, because of the long persistent length (50 nm) of double-stranded DNA^27^, when the length of DNA is short they are often treated as the rigid rods^23^,^28^. Besides, some surface beads carry the charge of −*e* to ensure that the linear charge density is about 1 e/0.17 nm^28^. Further, in order to depict the order degree of DNA orientation, we define the order parameter *S* by calculating the averaged value of DNA orientation with respect to *z* direction: 
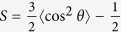
, where *θ* is the angle between DNA molecules and *z* direction. The thermo-responsive polymers that are grafted to the substrate and covalently bonded to DNA molecules^29^ are composed of several connected beads (the number of the beads can be varied, e.g., there are 20 beads per chain in Fig. 1), whose hydrophilicity/hydrophobicity changes with external temperature^30^.

### Thermo-responsive behaviors of DNA orientation in simulation

**Figure 2:**
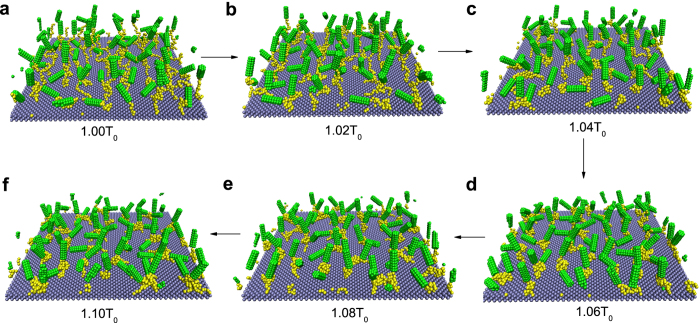
Snapshots of final equilibrium under different temperatures by using a general thermo-responsive polymer. (**a**) 1.00 *T*_0_, (**b**) 1.02 *T*_0_, (**c**) 1.04 *T*_0_, (**d**) 1.06 *T*_0_, (**e**) 1.08 *T*_0_, and (**f**) 1.10 *T*_0_. The temperature is gradually increased from 1.0 *T*_0_ to 1.1 *T*_0_.

As shown in Fig. 2a–d, when the temperature is low (1.0 *T*_0_), the polymers are totally hydrophilic and behave like random-walk chain, which induces the random distribution of their end-grafted DNA molecules. In addition, since the space for each DNA molecule under this situation is large due to long polymer length along *z* direction, the repulsive electrostatic interaction between DNA molecules is small and may have little effect on their orientations. Thus the DNA orientation is approximately random so that the order parameter *S* is low. As the temperature increases, the polymers become weakly hydrophobic, which will make them collapsed. As a result, the mean length in *z* direction becomes shorter and the space for DNA molecules becomes smaller. To minimize the electrostatic repulsive interaction as well as the excluded volume interactions, the orientation of DNA molecules will tend to be uniform and the order parameter *S* increases. But since the hydrophobicity is not very large, there still exist some polymers that are stretched by DNA molecules to decrease the electrostatic repulsive interaction (see snapshots of 1.02 *T*_0_ and 1.04 *T*_0_). Nevertheless, with a further increase in temperature, the polymers are almost collapsed into globules. We find that when the collapse of polymers becomes more obvious, the orientation should be more uniform, namely the order parameter increases with increasing the temperature. However, when the polymers become more and more hydrophobic, as shown in Fig. 2a–d (and Supplementary Figure 1), to avoid the contact with water, the neighboring polymers may aggregate and crosslink into domains. The averaged aggregation number which can quantitatively describe the crosslinking degree of polymers will increase with the hydrophobicity of polymers. Since the DNA is grafted to the polymers, the crosslinked polymers will enforce the corresponding DNA molecules close to each other. In this case, the uniform orientation of DNA molecules is not the most energy-favorable state. Instead, the DNA molecules grafted to the same polymeric domain may distribute like flowers (see Fig. 2e,f) to reduce the distance between each other, and the order parameter *S* is therefore decreased. Generally, the DNA orientation here shows triple-responsive behaviors, i.e, the order parameter is small under low- and high-temperature conditions while it becomes large when the temperature is in middle range.

**Figure 3:**
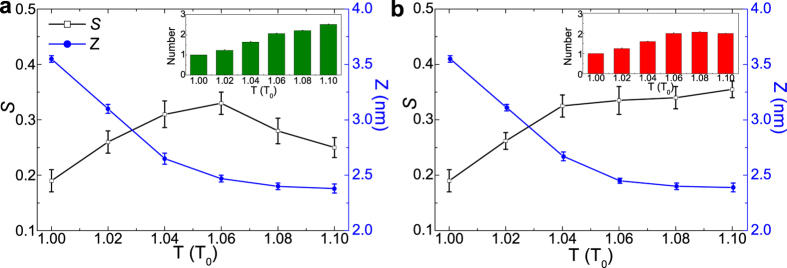
Physical parameters describing the responsive behaviors under two conditions. The order parameter *S*, z coordinate of center of mass (COM) of polymers (notice that z coordinate of the substrate is 1.0 nm), and (inset) averaged aggregation number as a function of temperature. (**a**) shows the case I where the temperature gradually increases from 1.0 *T*_0_ to 1.1 *T*_0_, whereas (**b**) shows the case II where the temperature sharply increases from 1.0 *T*_0_ to targeted temperature. The polymer length is 20.

Interestingly, we also notice that the dynamic process of increasing the temperature is also of great importance. Fig. 3a (case I) shows the case where the temperature gradually increases from 1.0 *T*_0_ to 1.1 *T*_0_ (i.e., the case discussed above) while Fig. 3b (case II) shows the case where the temperature sharply increases from 1.0 *T*_0_ to targeted temperature. By comparing Fig. 3a with Fig. 3b, little difference occurs when the temperature is low. But there exists notable difference under high temperature. This is due to the fact that the polymers will quickly collapse into globules in case II and the radius of globule is very small, which may weaken the crosslinking effect (see the inset in Fig. 3b). As a result, the order parameter will not decrease under this situation. Instead, due to the obvious collapse, it will increase a little with temperature. Actually, this difference again indicates that the obvious crosslinking behavior is the main reason of the decrease of order parameter *S* at high temperatures in the case I.

### Polymer length effect on DNA orientation in simulation

**Figure 4:**
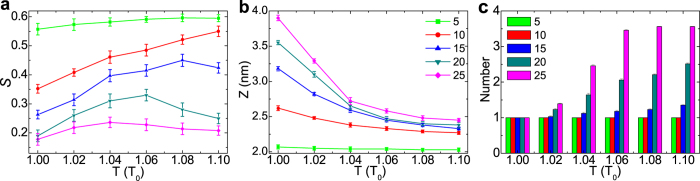
Physical parameters describing the responsive behaviors for different polymer lengths. (**a**) the order parameter *S*, (**b**) z coordinate of COM of polymers, and (**c**) averaged aggregation number as a function of temperature when the polymer length is 5, 10, 15, 20, and 25, respectively. The temperature gradually increases from 1.0 *T*_0_ to 1.1 *T*_0_.

Further, since the polymer length is one of the most important design parameters in experiments, here we also examine the effect of thermo-responsive polymer length on the DNA orientation. As shown in Fig. 4, for short polymers, the DNA order parameter changes very little with temperature. This is because the collapse is not obvious under this situation (i.e., z changes very little, see Fig. 4b), and the mean space for each DNA almost remains the same. With the increase of polymer length, the collapse of polymers under high temperature becomes obvious, which in turn causes the alignment of DNA molecules more consistently to minimize the excluded volume interaction and the repulsive electrostatic interaction. As a result, the orientational order increases with the increase of temperature. Further increase of polymer length may induce the crosslinking behavior at high-temperature regimes. As shown in Fig. 4c, there exist obvious crosslinking polymer domains when the polymer length is 20 and 25. Therefore, as discussed above, the order parameter will begin to decrease under high temperature. Additionally, when the length is sufficiently long, the crosslinking behavior will become obvious even if the temperature is not very high, which will suppress the increase of the DNA order parameter. Therefore under this situation (e.g., when the polymer length is 45, shown in Supplementary Figure 2a), the order parameter will change little and there does not exist the triple-responsive behaviors. Generally, on the basis of above discussion, we may choose the polymer length about 10 to generate dual-thermo–responsive behavior, and the length between 20 and 25 for triple-thermo-responsive behavior.

### PNIPAm length effect on DNA orientation in theory

**Figure 5:**
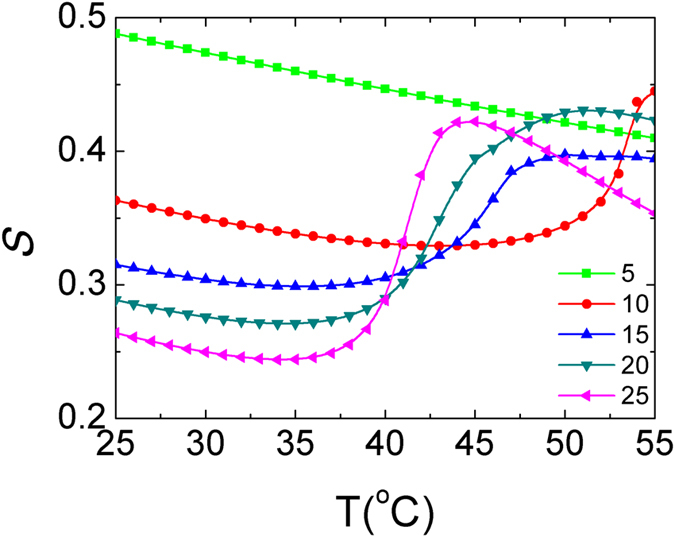
The DNA order parameter as a function of temperature under different PNIPAm lengths with the same surface coverage *σ* = 0.04 nm^−2^.

In order to obtain more physical insights of the thermo-responsive behaviors of DNA orientation, we further use the molecular theory to study the present system. In our theory, we use PNIPAm as the specific thermo-responsive polymer, and focus on the conditions of surface coverage *σ* = 0.04 nm^−2^ and PNIPAm length L = 5, 10, 15, 20, and 25 respectively in accordance with those used in simulations. In Fig. 5, we show the DNA order parameter as a function of temperature under five PNIPAm lengths. For short PNIPAm length (i.e., L = 5) system, the DNA order parameter linearly decreases with increasing the temperature without the appearance of obvious transition. For longer PNIPAm length (i.e., L = 10, 15) system, one obvious transition of the DNA order parameter takes place when the temperature increases to a critical value, which is called lower critical solution temperature (LCST). This transition is so sharp that it is finished within only a few degrees Celsius. For the system with L = 20, 25, in addition to the first sharp transition of DNA order parameter at LCST (here we should notice that the LCST of longer PNIPAm system is smaller than that of shorter PNIPAm system), a second transition appears at a higher temperature, where the DNA order parameter begins to decrease again. This means that the DNA order parameter under these two PNIPAm lengths shows a triple-thermo-responsive behavior. We conclude that the DNA orientation can show different thermo-responsive behaviors under different PNIPAm lengths.

### Physical mechanism of thermo-responsive behaviors

**Figure 6:**
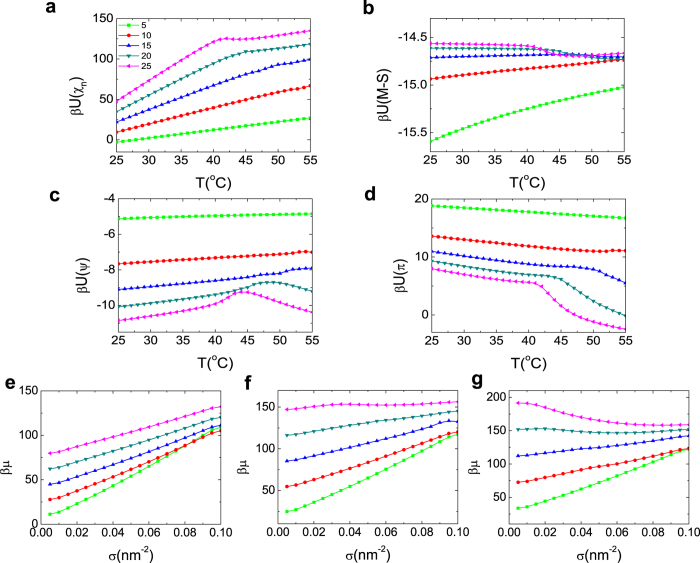
Physical mechanism of thermo-responsive behaviors. (**a**–**d**) The potential of mean force as a function of temperature. (**a**) PNIPAm hydrophilicity, (**b**) M-S interaction, (**c**) electric interaction, and (**d**) excluded volume interaction. (**e**–**g**) Chemical potentials of five different PNIPAm length systems under three different temperatures. (**e**) At 30 °C, all chemical potentials rise with the increase of *σ*, suggesting that all the systems are stable and no transitions occur; (**f**) At 42 °C, chemical potential of the system with L = 25 decreases with the increase of surface coverage in the region of 0.04 *nm*^−2^ < *σ* < 0.06 *nm*^−2^, meaning that the micro-phase separation occurs in this system; (**g**) At 50 °C, the micro-phase separation takes place not only in the systems with L = 25 under all surface coverages, but also in the systems with L = 20 in the region 0.02 *nm*^−2^ < *σ* < 0.05 *nm*^−2^. This means that the micro-phase separation will happen when the temperature is high, especially for the systems with longer PNIPAm. Parameters are the same as Fig. 5.

Since the DNA orientation can show different thermo-responsive behaviors, it is of great importance to reveal the underlying physical mechanism of these differences. Here we calculate the potential of mean force (PMF) and chemical potential under different PNIPAm lengths. In our system, there are mainly four kinds of driving forces. We calculate their PMF by *βU*(*i*) = −ln *q*(*i*), where i = *χ*, M-S, *ψ*, *π* represent PNIPAm hydrophobicity, Maier-Saupe interaction (i.e., the anisotropic attractive interaction of the rod-like dsDNA which is used to improve excluded volume interaction)^31^, electric interaction and excluded volume interaction, shown in Fig. 6a–d, respectively. The four kinds of PMF show different profiles as a function of temperature under short and long PNIPAm, which leads to the different thermo-responsive behaviors of DNA orientation. More specifically, for the system of L = 5, as the temperature increases, the hydrophobicity of PNIPAm molecules changes very little so that their conformations remain almost the same. Meanwhile, there is no change of space for DNA molecules. As a result, no transition happens to the DNA order parameter, and it just decreases a little due to the increase of copolymer conformational entropy as the temperature increases (see Supplementary Figure 3).

For longer PNIPAm system, transitions of the four kinds of PMF take place as the temperature increases, which further induces the transition of the DNA order parameter. Here we analyze the triple-thermo-responsive system (L = 25) concretely and focus on the two critical temperatures of 40 °C (LCST) and 45 °C. At low temperature, PNIPAm molecules are hydrophilic. They get hydrophobic as the temperature increases, and shrink a little due to the electrostatic repulsion between the DNA molecules. Therefore, the space for DNA molecules is large, and they are randomly distributed which results in the low order parameter. As the temperature rises close to LCST (40 °C), PNIPAm molecules get much more hydrophobic quickly and the electrostatic repulsion cannot prevent them from sharply collapsing onto substrate to reduce the contact with water. Meanwhile, the DNA molecules are dragged into a much smaller space. As a result, the electrostatic repulsion as well as the excluded volume interaction becomes stronger. The DNA molecules will get much ordered to balance out these interactions. As the temperature further increases and gets close to the second critical temperature (45 °C), PNIPAm molecules get more hydrophobic. This leads to the appearance of the micro-phase separation (see Fig. 6e–g), meaning that PNIPAm molecule domains have formed on the substrate. DNA molecules in domains are so crowded that they escape from each other due to the strong electrostatic repulsion, which results in the decrease of DNA order parameter (see Supplementary Figure 4).

### Comparison of simulation and theory results

As shown above, we use the DPD simulation and molecular theory to separately study the new designed thermo-responsive system, so it is necessary to compare the main results obtained by the two different methods. As a whole, the results agree well with each other, namely, for the system with L = 5, no DNA orientation transition takes place with the increase of temperature; for longer PNIPAm systems (L = 10, 15), the DNA orientation shows dual-thermo-responsive behaviors; for the systems with L = 20, 25, the DNA orientation shows triple-thermo-responsive behaviors. However, due to the different characters of thermo-responsive polymers used in simulation and theory (in simulation, we use general thermo-responsive polymers which get hydrophobic gradually with the increase of temperature; while in molecular theory, PNIPAm molecules get hydrophobic quickly and collapse sharply as the temperature gets close to their LCST), the transition of the DNA order parameter in theory is sharper than that in simulation. Moreover, the thermo-responsive degrees under different polymer lengths in simulation and theory are also slightly different (e.g., L = 25). Nevertheless, for the systems with very long polymer (L = 55), the results by simulations and theory also shows the same trend, i.e., the DNA orientation exhibits no thermo-responsive behaviors (see Supplementary Figure 2).

## Discussion

**Figure 7:**
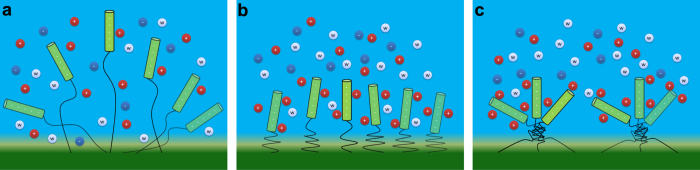
Three different status of the triple-responsive biosensor. (**a**) When the temperature is low, the polymer is hydrophilic and extends randomly, and the DNA order parameter is low; (**b**) When the temperature becomes high, the polymer gets hydrophobic and sharply collapses, and the orientaional DNA order is enhanced; (**c**) When the temperature is sufficient high, the micro-phase separation of polymers appears, reducing the orientational DNA order.

In the present study, we propose a new strategy to control the DNA orientation in biosensors and combine DPD simulation and molecular theory to prove its feasibility. Especially, when the thermo-responsive polymer has proper length, the DNA orientation is triple-responsive as shown in Fig. 7. The order parameter is low at low temperature. As the temperature increases, the DNA molecules get much ordered. But further increase of temperature leads to the decrease of the DNA order parameter. Furthermore, we point out the feasibility and application of our reported strategy in real detections. Firstly, we should notice that the present strategy can also be used to detect the binding of small molecules. In order to demonstrate this, we also investigate the DNA orientation as a function of temperature in the presence of protein, and find that the DNA order parameter becomes larger than that in the absence of protein (see Supplementary Figure 5). Due to the fact that the variation of DNA orientation can be detected by fluorescence energy transfer^16^, the detection of proteins may be achieved. Besides, when detecting charged molecules (e.g., proteins), the present strategy could be more suitable than electric-field method, since the electric field may confuse the binding of charged molecules. However, when compared with electric-field method, the variation degree of DNA orientation in our strategy is not so obvious, which may lead to a lower sensitivity of the detection. Moreover, small molecules may bind to thermo-responsive polymers^32^, which will also influence the sensitivity of the detection. These are beyond the scope of the present work and we will address the above issues in our future works.

Finally, we should notice that although thermo-responsive polymers are used in our study, the new strategy here is general because the responsive behavior of DNA orientation is just caused by swelling/deswelling transitions of polymers. Therefore, other types of stimuli-responsive polymers can also be used to take place of the thermo-responsive polymers in the system. For example, by using pH-sensitive polymers like PAH^20^,^33^, the pH-responsive detectiom can be fabricated, which may be well suited in specific organs (such as the gastrointestinal tract or the vagina) or intracellular compartments (such as endosomes or lysosomes); The light-responsive detection can be synthesized by using photo-sensitive polymer containing o-nitrobenzyl^21^,^34^, which may have the advantage of non-invasiveness and the possibility of remote spatiotemporal control. Therefore, our strategy here can enrich the methods of controlling DNA orientation. We expect that the new strategy for controlling DNA orientation could be engineered experimentally with the advance of present nanotechnology, and believe that it can promote future development on novel design of biosensors.

## Methods

We briefly summarize the simulation and theory method used in this work and the details can be found in Supplementary Information.

### Dissipative particle dynamics (DPD) simulation

Dissipative particle dynamics (DPD) is a coarse-grained simulation technique with hydrodynamic interaction^35^. The dynamics of the elementary units which are so-called DPD beads, is governed by Newton’s equation of motion. Typically, there are three types of pairwise forces in the DPD, i.e., the conservative force, dissipative force, and random force. Here, in order to include electrostatic interaction between charged beads, the Coulomb force is incorporated into our DPD simulations^36^,^37^. Additionally, we also use a harmonic bond to ensure the integrality of polymers^38^. All simulations are performed in the NVT ensembles using the velocity-Verlet integration algorithm. Since our simulations involve the change of temperature, we choose room temperature as the reference, i.e., setting *k*_*B*_*T*_0_ = 1.0, where *T*_0_ = 298 K. The reduced temperature can be defined as *T*^ *^ = *T*/*T*_0_^25^,^30^. The size of the simulation box is 40*r*_*c*_ × 40*r*_*c*_ × 40*r*_*c*_ with the number density of 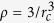. The integration time step Δ*t* = 0.015*τ* and each simulation time is at least 10^5^*τ*. All simulations in this work are carried out by using the modified soft package Lammps (12 Jun 2011)^39^.

### Molecular theory

The molecular theory was previously used to study the thermodynamics and structural properties of end-tethered neutral and charged polymers with the consideration of the conformation, size, and shape of each molecule^40^, and was shown to be in quantitative agreement with simulations and experimental observations^41^. More explicitly, the Helmholtz free energy per unit area for the systems in Fig. 1 is


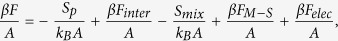


where *β* = 1/*k*_*B*_*T* is the inverse absolute temperature. *S*_*p*_ is the conformational entropy of copolymer, *F*_*inter*_ is the effective intermolecular interactions between PNIPAm segments and water, *S*_*mix*_ is the translational (mixing) entropy of small molecules, including the cations, anions and water, *F*_*M* − *S*_ describes anisotropic interactions for DNA in Mayer-Saupe self-consistent field approximation^42^, *F*_*elec*_ accounts for the electrostatic contribution to the free energy. Each of these terms of free energy can be explicitly expressed as a functional of probability of copolymer conformations, the local density profiles of the small molecules in the solution, the orientation of DNA and the electrostatic potential. Then by minimizing the free energy, a series of equations can be obtained, which can be solved by numerical method. All structural properties can be determined from the probabilities and interaction fields obtained from the minimized free energy, and any thermodynamic quantity of interest can be calculated. Technical details and all the relevant equations of the molecular theory can be found in the Supplementary Information.

## Additional Information

**How to cite this article**: Feng, C. *et al.* Designing new strategy for controlling DNA orientation in biosensors. *Sci. Rep.* **5**, 14415; doi: 10.1038/srep14415 (2015).

## Supplementary Material

Supplementary Information
