# Supplementary material for: Designing new strategy for controlling DNA orientation in biosensors
Source: Sci Rep. 2015 Sep 24;5:14415. doi: 10.1038/srep14415 (PMC4585838; doi:10.1038/srep14415)
Supplement: Supplementary Information [file srep14415-s1.pdf]

Supplementary Information for: Designing new strategy for controlling DNA orientation in biosensors

Chao Feng[1,2,†], Hong-ming Ding[1,2,†], Chun-lai Ren[1,2,*] & Yu-qiang Ma[1,2,3,*]

[1] *National Laboratory of Solid State Microstructures and Department of Physics, Nanjing University, Nanjing 210093, China*

[2] *Collaborative Innovation Center of Advanced Microstructures, Nanjing University, Nanjing, 210093, China*

[3] *Center for Soft Condensed Matter Physics and Interdisciplinary Research, Soochow University, Suzhou 215006, China*

[†] *These authors contributed equally to this work*

[*] *E-mail address: chunlair@nju.edu.cn, myqiang@nju.edu.cn*

I. SUPPLEMENTARY METHODS

1. DPD simulation method

The DPD is a coarse-grained simulation technique with hydrodynamic interaction[1]. The dynamics of the elementary units which are so-called DPD beads, is governed by Newton's equation of motion:

$$\frac{d\mathbf{r}_i}{dt} = \mathbf{v}_i; \quad \frac{d\mathbf{v}_i}{dt} = \mathbf{f}_i/m. \tag{1}$$

Typically, in the DPD, there are three types of pairwise forces acting on bead i by bead j: the conservative force, dissipative force, and random force. In the present work, the electrostatic force is introduced to take into account the electrostatic interactions between charged beads.

The conservative force $\mathbf{F}_{ij}^C$ is taken as

$$\mathbf{F}_{ij}^C = \begin{cases} a_{ij}(1 - r_{ij}/r_c)\hat{\mathbf{e}}_{ij} & r_{ij} < r_c \\ 0 & r_{ij} \geq r_c \end{cases}, \tag{2}$$

where $\mathbf{r}_{ij} = \mathbf{r}_i - \mathbf{r}_j, r_{ij} = |\mathbf{r}_{ij}|$, and $\hat{\mathbf{e}}_{ij} = \mathbf{r}_{ij}/r_{ij}$. The parameter r_c is the cutoff radius of the conservative force, and a_{ij} represents the maximum repulsion interaction of beads of type i and type j. For any two beads of the same type, we take the repulsive parameter $a_{ii} = 25$. In our system, the water (W) and ion (I), DNA (D), plane (N) beads are hydrophilic (the ion concentration is about 0.10 M), thus the interaction parameter between these beads is fixed to be 25, i.e., $a_{WI} = a_{WD} = a_{WI} = a_{WN} = a_{ID} = a_{IN} = a_{DN} = 25$[1]. In particular, the hydrophilic/hydrophobic property of thermo-responsive polymer (P) is related to external temperature. For the sake of simplicity, here we assume that its hydrophilic/hydrophobic property changes linearly with the temperature, i.e, $a_{PW} = a_{PI} = a_{PD} = a_{PN} = 25 + (T - T_0) * 500$. This relationship shows that when the temperature is low (e.g., T_0), the polymer is totally hydrophilic ($a_{PW} = 25$), while it becomes very hydrophobic ($a_{PW} = 75$) when the temperature is high (e.g., $1.1T_0$).

The dissipative force $\mathbf{F}_{ij}^D$ is

$$\mathbf{F}_{ij}^D = \begin{cases} -\gamma(1 - r_{ij}/r_c)(\hat{\mathbf{e}}_{ij} \cdot \mathbf{v}_{ij})\hat{\mathbf{e}}_{ij} & r_{ij} < r_c \\ 0 & r_{ij} \geq r_c \end{cases}, \tag{3}$$

where $\mathbf{v}_{ij} = \mathbf{v}_i - \mathbf{v}_j$ is relative velocity between beads i and j, and γ is the strength of friction.

Finally, the random force $\mathbf{F}_{ij}^{R}$ takes the form of

$$\mathbf{F}_{ij}^{R} = \begin{cases} \sqrt{2\gamma k_B T}(1 - r_{ij}/r_c)\zeta_{ij}\Delta t^{-1/2}\hat{\mathbf{e}}_{ij} & r_{ij} < r_c \\ 0 & r_{ij} \geq r_c \end{cases}. \quad (4)$$

Here, ζ_{ij} is a symmetric random variable with zero mean and unit variance, namely $< \zeta_{ij}(t) >= 0$ and $< \zeta_{ij}(t)\zeta_{kl}(t') >= (\delta_{ik}\delta_{jl} + \delta_{il}\delta_{jk})(\delta(t-t'))$. Δt is the time step of simulation.

Electrostatic interactions were incorporated into the DPD simulations by Groot[2]. Since soft potential in the DPD allows for the overlap between DPD beads, when the charged DPD beads are modeled, this can lead to the formation of artificial ion pairs and cause the divergence of the electrostatic potential. To avoid this problem, Groot chose to spread out the charges using the distribution[2]:

$$\rho_e(r) = \frac{3}{\pi {R_e}^3}(1 - r/R_e), r < R_e, \quad (5)$$

where R_e is the electrostatic smearing radius, and is typically set as 1.6 r_c.

Further, we also use a harmonic bond $U_s = k_s(1 - r_{i,i+1}/l_0)^2$ (here we choose $k_s = 64$ and l_0=0.5 r_c) between the neighboring beads to ensure the integrality of polymers. Our simulations apply the velocity-Verlet integration algorithm and the integration time step Δt=0.015 τ. In addition, we choose the cutoff radius r_c, bead mass m, energy k_BT_0 as the simulation units. All simulations are performed in the NVT ensembles. The size of the simulation box is 40 r_c×40 r_c×40 r_c with the number density of $\rho = 3/r_c^3$. Similar to previous studies, r_c is about 1.0 nm in our system[3,4]. The periodic boundary conditions are adopted in three directions.

2. Molecular theory and numerical solution

2.1. Molecular theory

We use a molecular theory to model temperature-sensitive DNA-b-PNIPAm copolymers which are end-grafted to a planar substrate and immersed in an aqueous solution of salt ions (0.10 M, which is close to physiological salt concentration). The theory was previously used to study the thermodynamics and structural properties of end-tethered neutral and charged polymers with the consideration of the conformation, size, and shape of each molecule[5–7], and was shown to be in quantitative agreement with simulations and experimental observations[8–10]. More explicitly, the Helmholtz free energy for the system in Fig. 1

of the main text is

$$\frac{\beta F}{A} = -\frac{S_p}{k_B A} + \frac{\beta F_{inter}}{A} - \frac{S_{mix}}{k_B A} + \frac{\beta F_{M-S}}{A} + \frac{\beta F_{elec}}{A}, \tag{6}$$

where $\beta = 1/k_B T$ is the inverse absolute temperature.

The first term in the right-hand side of Eq. 6 is the conformational entropy of copolymer chains, which is written as:

$$-\frac{S_p}{k_B A} = \sigma \sum_{\alpha} p(\alpha) \ln p(\alpha), \tag{7}$$

where σ is the surface coverage of copolymer and $p(\alpha)$ is the probability distribution function (pdf) of finding a copolymer in conformation α, and we can utilize it to calculate any thermodynamical and structural quantity of the copolymer as well as the orientation of DNA. For example, the polymer volume fraction is expressed by

$$\langle \phi_i(z) \rangle dz = \sigma \sum_{\alpha} p(\alpha) n_i(\alpha, z) v_i dz. \tag{8}$$

Here $n_i(\alpha, z)dz$ denotes the number of segments of type i that a copolymer chain in conformation α contributes in the layer between z and $z + dz$ ($i = n, D$ represent PNIPAm and DNA, respectively). v_i is the volume of each segment of type i.

The second term of Eq. 6 represents the effective intermolecular interactions between PNIPAm segments and water, which is

$$\frac{\beta F_{inter}}{A} = \int \frac{\chi_{nw}(z)}{v_w} \phi_n(z) \phi_w(z) dz, \tag{9}$$

where $\chi_{nw}(z)$ describe the strength of the PNIPAm-water effective repulsions as well as the association among PNIPAm monomers with the increase of temperature. The water volume fraction is given by $\phi_w(z) = \rho_w(z) v_w$, with the density of water molecules $\rho_w(z)$ in the layer between z and $z + dz$ and volume of water molecule v_w, which is used as the unit of volume.

The third term in free energy expression is the translational (mixing) entropy of small molecules, including the cations, anions and water. It is given by

$$\begin{aligned} -\frac{S_{mix}}{k_B A} = & \int \rho_{Na+}(z)[\ln \rho_{Na+}(z) v_w - 1] dz + \\ & \int \rho_{Cl-}(z)[\ln \rho_{Cl-}(z) v_w - 1] dz + \\ & \int \rho_w(z)[\ln \rho_w(z) v_w - 1] dz, \end{aligned} \tag{10}$$

where $\rho_i(z)$ is the density of molecule of species i.

The fourth term in Eq. 6 describes the anisotropic interactions for DNA in Mayer-Saupe self-consistent field approximation[11–13], which is as follows:

$$\frac{\beta F_{M-S}}{A} = \frac{1}{v_w} \int [-\frac{\eta}{2}\phi_D^2(z)s_2^2(z)]dz, \tag{11}$$

where $s_2(z)$ is the order parameter defined through an averaged value of the DNA orientation with respect to the director(i.e., z axis), and it is given by

$$s_2(z) = \frac{3}{2}\langle\cos^2\theta(\alpha,z)\rangle - \frac{1}{2} = \frac{3}{2}\frac{\sum_\alpha p(\alpha)n_D(\alpha,z)\cos^2\theta(\alpha,z)dz}{\sum_\alpha p(\alpha)n_D(\alpha,z)dz} - \frac{1}{2}, \tag{12}$$

where $\theta(\alpha,z)$ is the orientation of the DNA in conformation α in the layer from z to $z+dz$ with respect to z axis.

The last term in Eq. 6 accounts for the electrostatic contribution to the free energy, which is given by

$$\frac{\beta F_{elec}}{A} = \int [\langle\rho_q(z)\rangle\beta\psi(z) - \frac{1}{2}\varepsilon\beta(\nabla\psi(z))^2]dz, \tag{13}$$

where $\psi(z)$ is the local electrostatic potential, and ε is the dielectric constant of water. $\langle\rho_q(z)\rangle$ is the average charge density at z including contributions coming from all the charged species, which is given by

$$\langle\rho_q(z)\rangle = \rho_{Na+}(z)e + \rho_{Cl-}(z)(-e) + \rho_D(z)(-e), \tag{14}$$

where e is the elementary charge. Here we consider all charged small molecules, that is, Na^+, Cl^-. The last term comes from the contribution of DNA and each base pair with a charge of $-e$.

For an equilibrium state of the system, two constraints should be satisfied. One is the packing or incompressibility constraint, which reflects the intermolecular repulsions at each layer, namely

$$\phi_n(z) + \phi_D(z) + \rho_{Na^+}(z)v_{Na^+} + \rho_{Cl^-}(z)v_{Cl^-} + \phi_w(z) = 1. \tag{15}$$

The other constraint is the global electroneutrality, given by:

$$\int \langle\rho_q(z)\rangle dz = 0. \tag{16}$$

The packing and electroneutrality constraints are fulfilled by introducing the Lagrange multipliers $\pi(z)$ and λ into the free energy. Notice that the system is in contact with a bath of all the other species and therefore the calculations have to be performed in the semigrand canonical ensemble. By including two constraints, we write down the semigrand potential density:

$$\begin{aligned}\frac{\beta W}{A} &= \frac{\beta F}{A} - \beta\mu_{Na^+}\int \rho_{Na^+}(z)dz - \beta\mu_{Cl^-}\int \rho_{Cl^-}(z)dz + \beta\lambda\int \langle\rho_q(z)\rangle dz \\ &\quad + \beta\int \pi(z)[\phi_n(z) + \phi_D(z) + \phi_w(z) + \rho_{Na^+}(z)v_{Na^+} + \rho_{Cl^-}(z)v_{Cl^-} - 1]dz, \quad (17)\end{aligned}$$

where the chemical potentials for Na^+ and Cl^- are in reality exchange chemical potentials.

To find equilibrium solutions, we minimize the semigrand potential with respect to different variables. It turns out that the probability distribution function $p(\alpha)$ is expressed as:

$$\begin{aligned}p(\alpha) &= \frac{1}{q}\exp\{-\int \frac{\chi_{nw}(z)}{v_w} n_n(\alpha, z)v_n\phi_w(z)dz - \int \frac{\partial x_{nw}(z)}{\partial\phi_n(z)} n_n(\alpha, z)v_n/v_w\phi_n(z)\phi_w(z)dz \\ &\quad + \int \frac{\eta}{v_w}\phi_D(z)s_2(z)n_{ds}(\alpha, z)v_{ds}[\frac{3}{2}\cos^2\theta(\alpha, z) - \frac{1}{2}]dz \\ &\quad - \int \beta(\psi(z) + \lambda)(-e)n_{ds}(\alpha, z)dz \\ &\quad - \int \beta\pi(z)[n_n(\alpha, z)v_n + n_{ds}(\alpha, z)v_{ds}]dz\}. \quad (18)\end{aligned}$$

The volume fraction of water is given by:

$$\phi_w(z) = \exp[-\beta\pi(r)v_w - \chi_{nw}(z)\langle\phi_n(z)\rangle]. \quad (19)$$

The densities of Na^+ and Cl^- are:

$$\rho_{Na^+}(z)v_w = \exp[\beta\mu_{Na^+} - \beta(\psi(z) + \lambda)e - \beta\pi(z)v_{Na^+}], \quad (20)$$

$$\rho_{Cl^-}(z)v_w = \exp[\beta\mu_{Cl^-} - \beta(\psi(z) + \lambda)(-e) - \beta\pi(z)v_{Cl^-}]. \quad (21)$$

For the electrostatic potential, it gives rise to a generalized Poisson-Boltzmann equation:

$$\epsilon\nabla^2\psi(z) = -\langle\rho_q(z)\rangle, \quad (22)$$

with the boundary conditions:

$$\lim_{z\to+\infty}\psi(z) = 0. \quad (23)$$

For the neutral surface, it becomes:

$$\left.\frac{\partial\psi(z)}{\partial z}\right|_{z=0} = 0. \tag{24}$$

Given the fact that the system is immersed in the bulk solution, the chemical potentials of Na^+ , Cl^- and the Lagrange multiplier λ can be transformed as functions of the bulk concentration. Unknown parameters in the above equations are the lateral pressure $\beta\pi(z)$ and electrostatic potential $\psi(z)$. These quantities can be determined by substituting Eq. 18-21 into the packing constraint (Eq. 15) and the Poisson−Boltzmann equation (Eq. 22). In practice, we convert the integral equations into a set of coupled nonlinear equations by discretizing the space, whereas details on the discretization and numerical methodology and how the chains are generated can be found in the following.

2.2. Numerical solution

Here we present an outline of the numerical method used to solve the equations derived from the molecular theory. This is done by dividing the z-axis into parallel spherical layers of thickness δ. Functions are assumed to be constant within a layer; hence integrations can be replaced by summations. The ith layer is defined as the region between $(i-1)\delta \leq r < i\delta$. The packing constraints, Eq. 15, in a discrete form for layer i is:

$$\phi_n(i) + \phi_D(i) + \rho_{Na^+}(i)v_{Na^+} + \rho_{Cl^-}(i)v_{Cl^-} + \phi_w(i) = 1. \tag{25}$$

The volume fraction of PNIPAM and DNA equals

$$\langle\phi_j(i)\rangle = \sigma\sum_{\alpha} p(\alpha)n_j(\alpha,i)v_j(j=n,D). \tag{26}$$

Here the discretized probability distribution functions $p(\alpha)$ is

$$\begin{aligned} p(\alpha) \;=\; & \frac{1}{q}\prod_{i=1}^{i=i_{max}} \exp\{-\frac{\chi_{nw}(i)}{v_w}n_n(\alpha,i)v_n\phi_w(i) - \frac{\partial\chi_{nw}(i)}{\partial\phi_n(i)}n_n(\alpha,i)v_n/v_w\phi_n(i)\phi_w(i) \\ & +\frac{\eta}{v_w}\phi_D(i)s_2(i)n_{ds}(\alpha,i)v_{ds}[\frac{3}{2}\cos^2\theta(\alpha,i) - \frac{1}{2}] - \beta(\psi(i)+\lambda)(-ne)n_{ds}(\alpha,i) \\ & -\beta\pi(i)[n_n(\alpha,i)v_n + n_{ds}(\alpha,i)v_{ds}]\}. \end{aligned} \tag{27}$$

The volume fractions of cations, anions and water are given by:

$$\phi_{Na^+}(i) = \phi_{Na^+,bulk}\exp[-\beta(\pi(i)-\pi_{bulk})v_{Na^+} - \beta\psi(i)e], \tag{28}$$

$$\phi_{Cl^-}(i) = \phi_{Cl^-,bulk}\exp[-\beta(\pi(i)-\pi_{bulk})v_{Cl^-} - \beta\psi(i)(-e)], \tag{29}$$

$$\phi_w(i) = \exp[-\beta\pi(i)v_w - \chi_{nw}(i)\langle\phi_n(i)\rangle]. \tag{30}$$

The generalized Poisson equation is described as follows:

$$\epsilon[\frac{\psi(i+1) - 2\psi(i) + \psi(i-1)}{\delta^2}] = -\langle\rho_q(i)\rangle. \tag{31}$$

Here $\langle\rho_q(i)\rangle$ is the total charge density in layer i, which is

$$\langle\rho_q(i)\rangle = \rho_{Na^+}(i)e + \rho_{Cl^-}(i)(-e) + \rho_D(i)(-e), \tag{32}$$

where $\rho_D(i)$ is

$$\langle\rho_D(i)\rangle = \sigma\sum_\alpha p(\alpha)n_D(\alpha, i). \tag{33}$$

The discrete boundary conditions are

$$\psi(1) - \psi(0) = 0, \tag{34}$$

and

$$\psi(n+1) = 0. \tag{35}$$

The discretized packing constraint and Poisson equation constitute a set of coupled nonlinear equations for $\pi(i)$ and $\psi(i)$. These coupled equations can be solved with standard numerical techniques.

The chain model for PNIPAm is the three-state RIS model[14]. In this model, each bond has three different isoenergetic states. The DNA is treated as rod, whose orientations are generated randomly. The conformations of DNA-b-PNIPAm are generated by a simple sampling method and all the accepted conformations are self-avoiding. We generate 10^6 independent conformations. In our calculations, each segment of PNIPAM has the volume of v_n=0.16 nm^3, which was chosen according to the partial specific volume of PNIPAM in water, and for water v_w=0.03 nm^3 as we use before[15]. the DNA is consisted of 12 base pair, each one has a negative charge (-e) and a volume of 1.0 nm^3. The layer thickness was σ=0.6 nm. The Maier-Saup parameter as a function of temperature for DNA is $\eta = 180.0/T - 0.45$[16,17]. The interaction parameter $\chi_{wn}(i) = (g_{00} + g_{02}T) + (g_{10} + g_{12}T)\phi_n(i) + (g_{20} + g_{22}T)\phi_n^2(i)$ between water and PNIPAM comes from the experiment of Afroze et al[18], where g_{ij} (i,j=0, 1, 2) are constants.

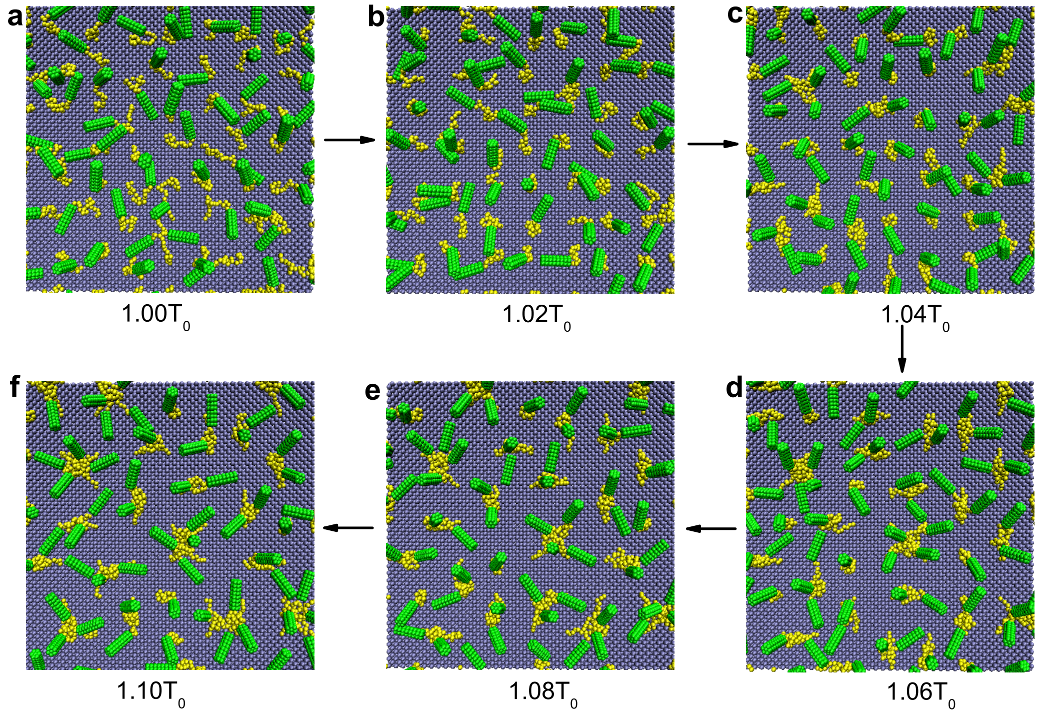

Supplementary Figure 1: Top view of snapshots of final equilibrium under different temperatures. (a) 1.00 T_0, (b) 1.02 T_0, (c) 1.04 T_0, (d) 1.06 T_0, (e) 1.08 T_0, and (f) 1.10 T_0. The temperature is gradually increased from 1.0 T_0 to 1.1 T_0.

II. SUPPLEMENTARY FIGURES AND DISCUSSIONS

Supplementary Fig. 1 shows the snapshots of final equilibrium of the system evolving with temperature in DPD simulations from top view. When the temperature increases, the polymers get more hydrophobic, and the crosslinking behavior between neighboring DNA molecules becomes more and more obvious, causing the flower-like distribution of DNA molecules under high temperatures.

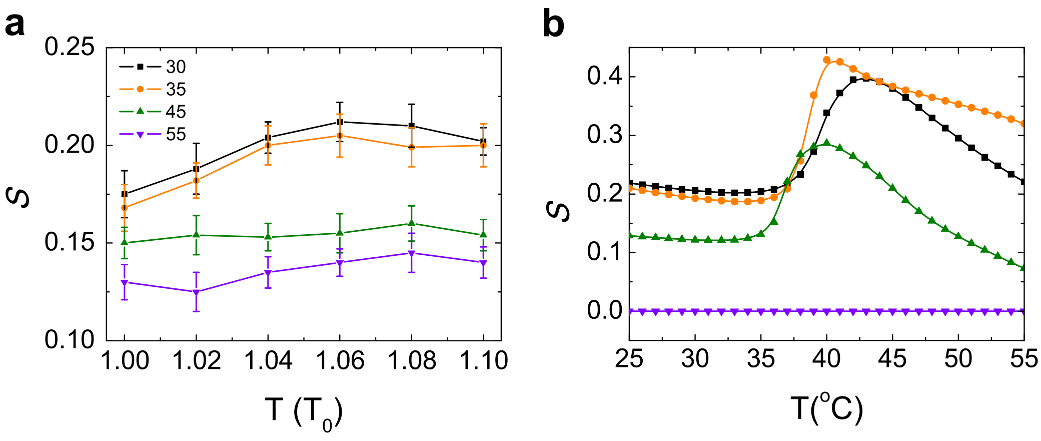

Supplementary Figure 2: Average order parameter of DNA as a function of temperature with long lengths of thermo-responsive polymer. (a) General polymer system in DPD simulation and (b) PNIPAm system in molecular theory. Squares, circles, triangles(up), triangles(down), correspond to $L = 30, 35, 45$, and 55, respectively. All cases are with the surface coverage σ=0.04 nm^{-2}.

Supplementary Fig. 2 shows the average order parameter of DNA in different systems with different lengths of thermo-responsive polymer. As we can see, triple-thermo-responsive behaviors get weakened with the increase of polymer length. Especially in the system with polymer length of 55, the responsive behaviors disappear.

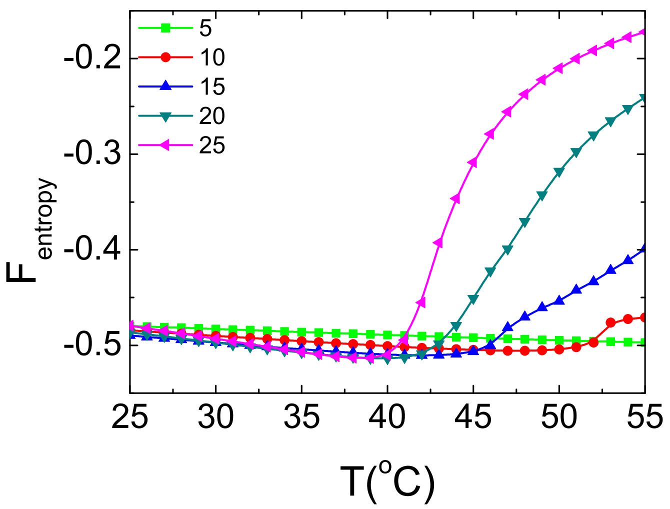

Supplementary Figure 3: Free energy of conformational entropy under different temperatures with the surface coverage σ=0.04 nm^{-2}. Squares, circles, triangles(up), triangles(down), triangles(left) correspond to $L = 5, 10, 15, 20$, and 25, respectively.

Supplementary Fig. 3 shows the free energy originating from copolymer conformational entropy under different temperatures. When the temperature is lower than 40 ^oC, as the temperature increases, the free energy of polymer conformational entropy decreases (i.e., the polymer conformational entropy increases), and reduces the orientational order of DNA molecules.

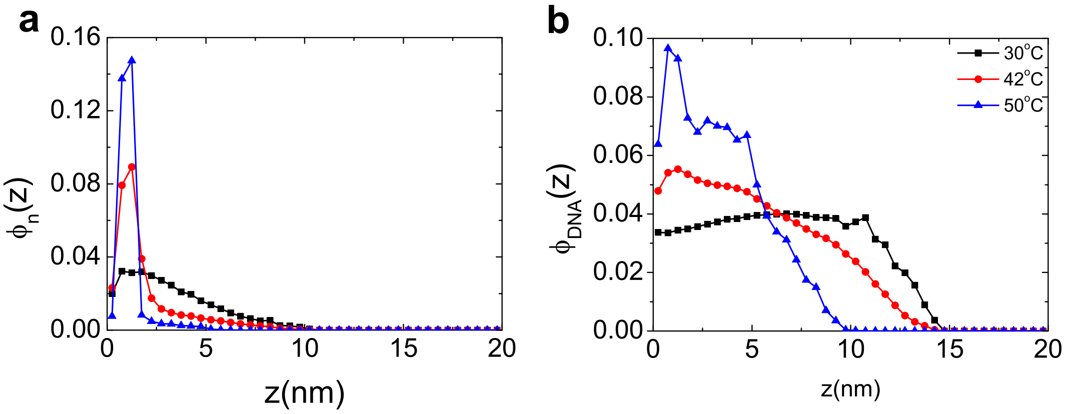

Supplementary Figure 4: Volume fraction distributions of (a) PNIPAm and (b) DNA as a function of the distance from the substrate under different temperatures with σ=0.04 nm^{-2} and $L = 25$. The squares, circles and triangles represent T=30, 42, and 50 ^oC, respectively.

Supplementary Fig. 4 shows the volume fraction of DNA and PNIPAm at the temperature of 30 ^oC, 42 ^oC and 50 ^oC, respectively. When the temperature is 30 ^oC, PNIPAm molecules are hydrophilic and swell freely from the surface to z=10 nm; DNA molecules randomly distribute from the surface to z=15 nm. As the temperature increases to 42 ^oC, PNIPAm molecules get hydrophobic and collapse sharply, focusing on 0-2.5 nm; Meanwhile, the DNA molecules are dragged close to substrate by PNIPAm and get close to each other. Here we should notice that the profile of DNA is linearly distributed from 5-15 nm, meaning that the ordered DNA molecules are not in the same layer, but layer by layer. As the temperature further increases to 50 ^oC, PNIPAm molecules get more hydrophobic, leading to the appearance of micro-phase separation of polymers. The DNA molecules are repulsed, and some of them even fall down onto the substrate.

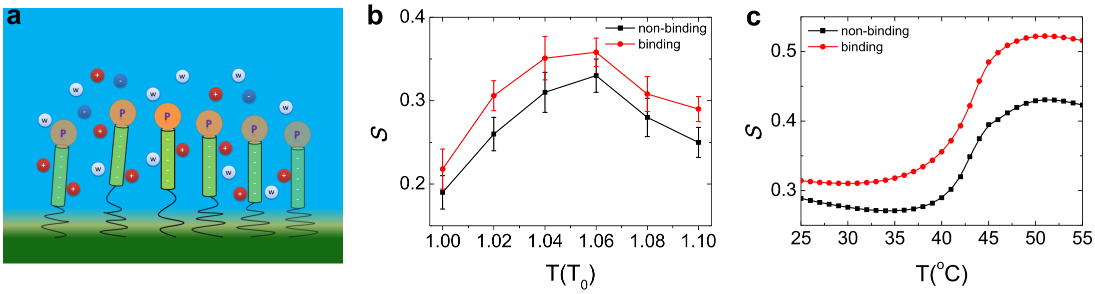

Supplementary Figure 5: The DNA order parameter as a function of temperature in the presence/absence of proteins. (a) Schematic illustration of our system in the presence of proteins (orange spheres marked with "p"), which are bound to the dsDNA's upper ends. (b) and (c) are the DNA order parameter without (squares) and with protein bindings (circles) derived from DPD simulation and molecular theory, respectively. The conditions are σ=0.04 nm^{-2} and $L = 20$.

Supplementary Fig. 5 shows the further investigation of DNA orientation as a function of temperature in the presence of proteins, which are treated as nanospheres in our system for the sake of simplicity[19]. Here we choose ubiquitin (Ub) with a diameter of 2 nm as an example, which is usually used in protein detection[20]. Both the DPD simulation and molecular theory results show that the DNA order parameter is larger than that in the absence of ubiquitin. Considering that the variation of DNA orientation can be detected experimentally by fluorescence energy transfer[21], thus the detection of protein can be achieved.

[1] Groot, R. D. & Warren, P. B. Dissipative particle dynamics: Bridging the gap between atomistic and mesoscopic simulation. *J. Chem. Phys.* **107**, 4423–4435 (1997).

[2] Groot, R. D. Electrostatic interactions in dissipative particle dynamics-simulation of polyelectrolytes and anionic surfactants. *J. Chem. Phys.* **118**, 11265–11277 (2003).

[3] Ding, H.-m. & Ma, Y.-q. Design maps for cellular uptake of gene nanovectors by computer simulation. *Biomaterials* **34**, 8401–8407 (2013).

[4] Yong, X., Kuksenok, O., Matyjaszewski, K. & Balazs, A. C. Harnessing interfacially-active nanorods to regenerate severed polymer gels. *Nano Lett.* **13**, 6269–6274 (2013).

[5] Szleifer, I. & Carignano, M. A. Tethered polymer layers. *Adv. Chem. Phys.* **94**, 165–260 (1996).

[6] Szleifer, I. & Carignano, M. A. Tethered polymer layers: phase transitions and reduction of protein adsorption. *Macromol. Rapid Commun.* **21**, 423–448 (2000).

[7] Nap, R., Gong, P. & Szleifer, I. Weak polyelectrolytes tethered to surfaces: Effect of geometry, acid-base equilibrium and electrical permittivity. *J. Polym.Sci. Pol. Phys.* **44**, 2638–2662 (2006).

[8] Ren, C.-l., Nap, R. J. & Szleifer, I. The role of hydrogen bonding in tethered polymer layers. *J. Phys. Chem. B* **112**, 16238–16248 (2008).

[9] Shvartzman-Cohen, R., Ren, C.-l., Szleifer, I. & Yerushalmi-Rozen, R. An isotopic effect in self-assembly of amphiphilic block copolymers: the role of hydrogen bonds. *Soft Matter* **5**, 5003–5011 (2009).

[10] Feng, C., Ren, C.-l. & Ma, Y.-q. The coexisting phase behavior of thermo-responsive copolymer solutions. *Soft Matter* **10**, 5523–5531 (2014).

[11] Maier, W. & Saupe, A. Eine einfache molekular-statistische theorie der nematischen kristallinflussigen phase .1. *Naturforsch. A* **14**, 882–889 (1959).

[12] Amoskov, V. M. & Birshtein, T. M. Polydisperse anisotropic brushes. *Macromolecules* **34**, 5331–5341 (2001).

[13] Mercurieva, A. A., Birshtein, T. M. & Amoskov, V. M. Theory of liquid-crystalline ordering in polymer brushes. *Macromol. Symp.* **252**, 90–100 (2007).

[14] Ren, C.-l. & Ma, Y.-q. Loading oligonucleotides on a nanoparticle regulated by a grafted polyethylenimine layer. *Soft Matter* **7**, 10841–10849 (2011).

[15] Kujawa, P. & Winnik, F. M. Volumetric studies of aqueous polymer solutions using pressure

perturbation calorimetry: A new look at the temperature-induced phase transition of poly(n-isopropylacrylamide) in water and d2o. *Macromolecules* **34**, 4130–4135 (2001).

16 Ho, V., Boudouris, B. W. & Segalman, R. A. Tuning polythiophene crystallization through systematic side chain functionalization. *Macromolecules* **43**, 7895–7899 (2010).

17 Lin, S.-H., Wu, S.-J., Ho, C.-C. & Su, W.-F. Rational design of versatile self-assembly morphology of rod-coil block copolymer. *Macromolecules* **46**, 2725–2732 (2013).

18 Afroze, F., Nies, E. & Berghmans, H. Phase transitions in the system poly(n-isopropylacrylamide)/water and swelling behaviour of the corresponding networks. *J. Mol. Struct.* **554**, 55–68 (2000).

19 Szleifer, I. Protein adsorption on surfaces with grafted polymers: A theoretical approach. *Biophys. J.* **72**, 595–612 (1997).

20 Langer, A. *et al.* Protein analysis by time-resolved measurements with an electro-switchable dna chip. *Nat. Commun.* **4**, 2099 (2013).

21 Knezevic, J. *et al.* Quantitation of affinity, avidity, and binding kinetics of protein analytes with a dynamically switchable biosurface. *J. Am. Chem. Soc.* **134**, 15225–15228 (2012).
